# GTP energy dependence of endocytosis and autophagy in the aging brain and Alzheimer’s disease

**DOI:** 10.1007/s11357-022-00717-x

**Published:** 2023-01-09

**Authors:** Ricardo A. Santana Martínez, Priyanka D. Pinky, Benjamin A. Harlan, Gregory J. Brewer

**Affiliations:** 1 Department of Biomedical Engineering, University of California Irvine, Irvine, CA 92697 USA; 2 Center for Neurobiology of Learning and Memory, University of California Irvine, Irvine, CA 92697 USA; 3 MIND Institute, University of California Irvine, Irvine, CA 92697 USA

**Keywords:** GTP, Energetics, Autophagy, Mitophagy, Endocytosis, Lysosomes, Alzheimer’s, Aging

## Abstract

Increased interest in the aging and Alzheimer’s disease (AD)-related impairments in autophagy in the brain raise important questions about regulation and treatment. Since many steps in endocytosis and autophagy depend on GTPases, new measures of cellular GTP levels are needed to evaluate energy regulation in aging and AD. The recent development of ratiometric GTP sensors (GEVALS) and findings that GTP levels are not homogenous inside cells raise new issues of regulation of GTPases by the local availability of GTP. In this review, we highlight the metabolism of GTP in relation to the Rab GTPases involved in formation of early endosomes, late endosomes, and lysosomal transport to execute the autophagic degradation of damaged cargo. Specific GTPases control macroautophagy (mitophagy), microautophagy, and chaperone-mediated autophagy (CMA). By inference, local GTP levels would control autophagy, if not in excess. Additional levels of control are imposed by the redox state of the cell, including thioredoxin involvement. Throughout this review, we emphasize the age-related changes that could contribute to deficits in GTP and AD. We conclude with prospects for boosting GTP levels and reversing age-related oxidative redox shift to restore autophagy. Therefore, GTP levels could regulate the numerous GTPases involved in endocytosis, autophagy, and vesicular trafficking. In aging, metabolic adaptation to a sedentary lifestyle could impair mitochondrial function generating less GTP and redox energy for healthy management of amyloid and tau proteostasis, synaptic function, and inflammation.

## GTP, the other energy currency, in aging and AD

Synthesis of the high-energy molecules ATP and GTP both rely on the redox state of NAD^+^/NADH and their sufficient concentrations. ATP synthesis from glycolysis is primarily powered by the oxidative power of NAD^+^ acting on reducing sugars. ATP synthesis by oxidative phosphorylation in mitochondria is powered by NADH generation in the TCA cycle for reduction of pyruvate, glycerol, or fatty acids with oxygen as the terminal electron acceptor in the electron transport chain. Our label-free studies in live rat or mouse hippocampal neurons indicate an age-related depletion of NAD and NADH, that could impair synthesis of ATP and GTP [1–4]. This depletion was further exacerbated in neurons from mice carrying transgenes for human beta amyloid and tau. Further, postmortem analysis of TCA cycle enzymes indicated up to 40% impairment in the TCA cycle enzymes from AD brains compared to age-matched controls [5]. Thus, age-related energetic depletion of NAD^+^ and NADH on top of AD-related mitochondrial impairment in the activity of TCA enzymes could locally affect ATP and GTP levels, even if their bulk concentrations were minimally affected.

The energy available from GTP hydrolysis is the same as ATP hydrolysis, but GTP is utilized for different purposes than ATP due to the selectivity of specific enzymes. GTP concentrations in cells are on average tenfold lower than the millimolar ATP. GTP is a major regulator of multiple energy-dependent cellular processes of protein synthesis and vesicular trafficking involving endocytosis and autophagy. The estimated total cellular GTP concentration in mammalian cells is in the range of 250–700 μM [6], but free GTP in cancer cell protrusions is closer to 30 μM [7]. Major proteins powered by GTP include dynamins for membrane fission and fusion, the small regulatory GTPases, and microtubules as detailed below. Beside de novo synthesis from inosine by IDMPH2 and xanthosine by GMPS, cellular sources of GTP are primarily localized nucleoside diphosphate kinases (NDPKs) from the non-metastatic genes (NME; sometimes called NM23) that convert ATP to GTP [8]. There are at least ten members of the NME gene family but only the first four have nucleoside diphosphate kinase activity. According to the Allen Brain Atlas, NME1 through 4 are strongly and selectively expressed in the mouse and human hippocampus where memory is impaired in AD. NME1–3 are cytoplasmic, while NME4 is mitochondrial. Since both distinct processes of endocytosis and autophagy are affected in aging and AD, we posit that alterations upstream affect both, possibly the age and AD-related consequence of limiting GTP levels on these amyloid-critical functions.

## Measurement of GTP levels

**Fig. 1:**
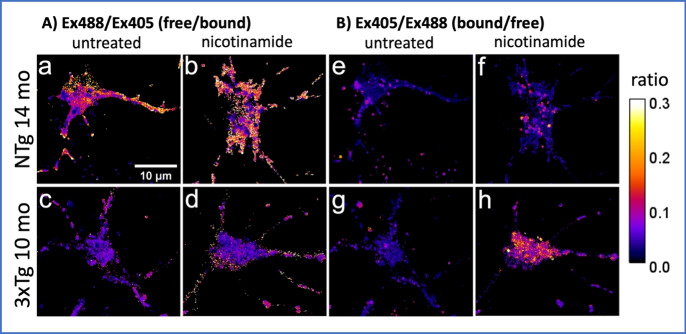
Ratiometric GTP-measurements in primary mouse hippocampal neurons from middle-age mice transfected with GEVAL530 sensor False-colored pixel-by-pixel ratiometric images of neurons show non-uniform distributions of GTP. (**a**) Excess free-GTP/bound-GTP of middle-age (14 mo.) non-transgenic (NTg) at edges and apical dendrite compared to (**c**) middle-age (10 mo.) 3xTg-AD neuron. (**b**, **d**) Increase in free GTP in neurons treated with 2 mM nicotinamide by 24 h. (**e**, **g**) Untreated neurons exhibit vesicular bound GTP that is increased in (**f**, **h**) neurons treated with 2 mM nicotinamide by 24 h

Although GTP regulates several crucial processes for cellular survival, measuring GTP levels in the live cell under physiological and pathological conditions is difficult because of high turnover and existence of both a protein-bound and free state. Bianchi-Smiraglia et al. [9] described a novel genetically encoded GTP sensor (GEVAL) based on yellow fluorescent protein that detects ratiometric changes in GTP-free and GTP-bound concentration in vitro and in vivo. Recently, the same group reported a mechanism for regulation of GTPase Rac1 in cell invasion by a human melanoma cell line driven by local GTP production [7] and reviewed their methodology [10]. As we will see in this review, GTP plays a crucial role during endocytosis and autophagy related to amyloid processing. Currently, we are studying the age-related GTP changes in processing of Aβ in primary cultures of hippocampal neurons from the triple transgenic 3xTg-AD mouse and its effect on alterations in autophagy. Figure 1 shows preliminary results of GEVAL530 transfection into primary adult neurons from this AD mouse model compared to non-transgenic mouse neurons. As observed by Bianchi-Smiraglia et al. [7], we see a non-uniform distribution of GTP in the processes and edges of the soma when measuring free GTP (Fig. 1Aa), with lower free GTP levels in the 3xTg-AD neurons (Fig. 1Ac). As detailed in Section 10, pretreatment of these neurons with an NAD^+^-precursor, nicotinamide, raised the free GTP levels (Fig. 1Ab, d). The 530-µM Kd for GEVAL530 used in these experiments suggests that the local free GTP concentration is within a few-fold of 530 µM. In Fig. 1B, we examined the bound GTP that appears to be localized in vesicles, and increased by nicotinamide (Fig. 1Bf, h), especially in the 3xTg-AD neurons. Preliminary evidence indicates that neurons treated with NME1 siRNA lowers their free GTP levels (Santana Martinez, unpublished). Further studies with adult neurons across the age-spectrum will determine the age-dependence of GTP deficits and the ability to remediate them with NAD^+^-precursors. Given the essential role of GTP in vesicular trafficking processes of endocytosis and autophagy and this preliminary evidence for changes in GTP with AD-like genetics, the following sections provide details of these processes and the effects of aging and Alzheimer’s disease on them. The result of this review highlights the need for new methods like GEVAL probes to directly measure changes in bound and free GTP and total GTP concentrations.

## Endocytosis: GTP dependence in aging and AD

**Fig. 2:**
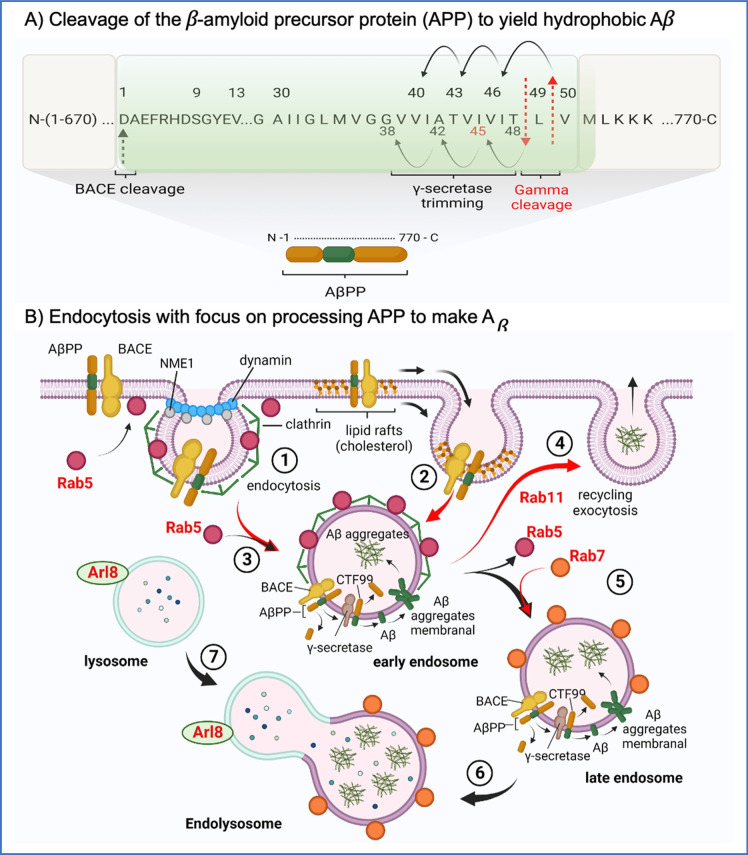
Endocytosis of the amyloid precursor protein (APP). **A** The N-terminal portion of the 770 residue APP protein with green shading of the aggregation-prone Aβ requiring cleavage by BACE and Gamma peptidases. **B** Endocytosis of APP. (1) Formation of the inward bud from the plasma membrane orchestrated by clathrin with endoproteases BACE and $\gamma$-secretase. Endocytosis supported by dynamin receives local GTP by NME1. (2) Microdomains enriched in cholesterol (lipid rafts) bind APP processed to Aβ or Aβ adsorbed from the parenchyma. (3) Early endosome attracts the GTPase Rab5 which is recognized by another GTPase, Rab11, to mediate (4) endocytic recycling of receptors back to the plasma membrane. Alternatively, (5) Rab7 mediates segregation early into late endosomes allowing further processing of APP and accumulation of Aβ. (6) Late endosome fusion with (7) lysosome to degrade contents. Some aggregated Aβ may be resistant to digestion, and with endosome disruption Aβ may accumulate in the cytosol

In the endocytosis process, the cell internalizes macromolecules and ligand-bound receptors and surface proteins including the amyloid precursor protein (APP) [11] (Fig. 2A). Of the two main endocytic pathways, plasma membrane-embedded APP uptake occurs mostly through clathrin-mediated uptake (Fig. 2B.1) rather than caveola-mediated from cholesterol-rich patches (Fig. 2B.2). However, hydrophobic Aβ, partitions into cholesterol-rich patches of lipid rafts in the plasma membrane with subsequent uptake by the caveola pathway [12] (Fig. 2B.2). In both cases, dynamin GTPase complexes assemble at the invagination to catalyze GTP-dependent membrane curvature for final fission and release of mature vesicles from the plasma membrane [13–15]. Local GTP fueling is catalyzed by NME1 and 2 nucleotide phosphate kinases that bind to dynamin [16].

### Early endosome Rab5 GTPase decision toward exocytosis and receptor recycling or transformation to late endosome Rab7 GTPase for degradation

The Rab5 GTPase is considered a marker for the early endosome [17]. Rab5 protein is present in the plasma membrane, in clathrin-coated vesicles, and early endosomes (Fig. 2). In cell and fly models of Huntington’s disease, Rab5 participates in autophagosome formation to regulate autophagy and eliminate toxic mutant huntingtin [18]. Inhibition of Rab5 reduces autophagy-regulating proteases Atg5-Atg12 conjugation, resulting in decreased autophagosome formation. The Atg12-Atg5 conjugate promotes lipidation of Atg8, and lipidated Atg8 facilitates autophagosome formation and selective cargo recognition during autophagy [19].

Another contributing factor to Rab5-mediated autophagy is phosphatidylinositol-3-kinase (PI3K). PI3-kinase complex inhibits autophagy by activating the Akt/mTOR-signaling pathway [20]. The P110β subunit of the PI3K complex regulates the catalytic activity of the Vps34 complex to promote PI3P generation [21]. This step is essential for autophagosome formation. P110β promotes the transition from Rab5-GDP to Rab5-GTP. P110β overexpression mitigates autophagic deficiency following activation of the macromolecular complex composed of Rab5, Vps34, and Beclin1, which in turn leads to autophagosome formation [22]. Targeting these specific proteins may elucidate therapeutic avenues to attenuate AD pathology.

Membrane-bound Rab5 is a key factor to directly promote Mon1-Ccz1 dependent Rab7 activation and Rab7-dependent membrane fusion [23]. Mon1-Ccz1 is a heterodimeric guanine nucleotide exchange factor GEF-complex which activates Rab5. Mon1-mediated displacement of the Rab5 GEF results in displacement of Rab5 by Rab7. C‐Vps complex acts as a GEF for Rab7 and promotes Rab7 transiting from the GDP‐bound to the GTP‐bound state for Rab7 activation. Ultraviolet radiation resistance–associated gene (UVRAG) stimulates Rab7 activation by UVRAG‐C‐Vps interaction through GDP/GTP exchange of Rab7 [24]. Another regulatory point in the autophagic process is homotypic fusion and vacuole protein‐sorting (HOPS) complex that activates the yeast vacuolar Ypt‐Rab GTPase during membrane fusion [25]. One of the 6 subunits of HOPS complex is the Class C vacuolar protein sorting (C‐Vps) complex which contains Vps11, Vps16, Vps18, and Vps33. Rab7 late endocytic vesicles subsequently fuse with lysosomes for cargo degradation (Fig. 2).

### Changes in Rab 5 and Rab 7 endosomal trafficking in mouse models and in AD

In mouse models of AD with mutations in the amyloid precursor protein (APP) or Aβ-producing presenilin, the endosome–autophagosome–lysosome pathway appears dysregulated partly because of impaired acidification of lysosomes that fails to sufficiently activate protease and lipases [26]. In mouse models of AD, large perinuclear bodies containing Aβ aggregates label with the autophagosome marker LC3 colocalized with Rab7 and late endosomal and lysosomal components. This could be due to failed protective upregulation or pathologic impairment. In neurons cultured from 3xTg-AD mice across the age-span, we found aggregated vesicular Aβ to increase 30–50 fold with age, most prominently in Rab5-labeled early endosomes and mitochondria, but also within Rab7-labeled late endosomes and autophagosomes [27]. The snow-ball-like accumulation of Aβ42 and Aβ45 suggests that old neurons were unable to complete autophagic degradation of these longer aggregates of Aβ. We hypothesize that impaired energy production in old neurons limits the energetic capacity for completion of autophagy. Compared to levels in young hippocampus and cortex, in 7–12-month-old APP/PS1 mouse hippocampus, Rab7 levels decreased along with reductions in Beclin1 and Rubicon activators and an increase in the Rubicon inhibitor [28]. In younger hippocampus and all ages in cortex, Beclin1 activates Rab7, while Rubicon inhibits the activation of Rab7 via suppression on UV radiation resistance-associated gene protein (UVRAG)‐vacuolar protein sorting gene (Vps) interaction. Rab7 deficiency in yeast and fruit flies results in massive accumulation of autophagosomes [29]. Rab7 knockdown also inactivates mTORC1/S6K1, and localization of mTOR in late endosomes [30]. Interestingly, the inhibition of other stages of endocytic trafficking does not change the activity of mTORC1 suggesting that intact late endosomes are crucial for mTORC1 signaling in autophagy. This is an important reason why targeting mTOR may not slow aging or AD.

Regionally selective upregulation of Rab 5 and Rab7 proteins and mRNAs in AD, suggests selective contributions to disease pathology or an insufficient protective mechanism [31]. Aβ accumulates predominantly in Rab7‐positive late endosomes and autophagic vacuoles of neuronal cells [32] in an age-related manner [27] causing internalization of Aβ and subsequent increase of Rab7 triggering neuronal degeneration [33]. Blocking the late endocytic pathway by Rab7 suppression induces Aβ‐dependent amyloid fibril formation on the cell surface causing endosomal enlargement [34] resulting in accelerated recycling of Rab7 and endocytic trafficking of Aβ to lysosomes for degradation [35]. Inhibition of Iysosomal proteolysis can also affect the axonal retrograde transport of autophagic organelles causing AD‐like axonal dystrophy [36]. However, practical interventions to promote Rab7 mediated autophagy could delay or reverse AD progression. Examples include a shift from a sedentary state to exercise, Mediterranean diet, and energy boosting compounds like NAD precursors to create a redox shift [4] (Section 10). Another avenue is the strange discovery that guanosine monophosphate reductase 1 (NADPH-dependent GMPR1) levels are increased in AD brains that could itself lower GTP levels and raise AMP signaling [37]. Neurofibrillary tangles of tau were lowered when this activity was inhibited in AD-mice.

With progression from mild cognitive impairment to AD, both endosomal Rab5 and Rab7 expression were upregulated in hippocampal CA1 neurons as measured by transcriptional microarray [31]. Enlargement of Rab5-positive endosomes was associated with neurofibrillary tangles and amyloid deposition. Neurotrophic factors such as nerve growth factor (NGF) bind to their Trk receptors and internalize into Rab5-positive endosomes to initiate downstream signaling [38]. It is interesting to note that, the signaling endosome is retained as Rab5 early endosomes, and does not progress to Rab7 late endosomes, during their transit within the long axons [39]. This differentiates receptor-mediated signal delivery from endosome processing toward autophagy [40]. Following activation by NGF, TrkA recruits a Rab5‐GAP to quickly convert GTP‐Rab5 to GDP‐Rab5 to keep the level of GTP‐Rab5 in check, which prevents the Rab5 to Rab7 conversion resulting in inhibition of the NGF/TrkA signaling and premature degradation.

Rab5 function is compromised in early phases of AD [41]. Persistent hyperactivation of Rab5 promoted the endocytic pathway toward late endosomes, lysosome fusion, and autophagy resulting in premature degradation of the neurotrophic factor signaling and neuronal atrophy. Vps35 and Vps26, two key retromer proteins, were also reduced in AD brains [42]. Vps26 binds to SorLA which is a sorting receptor that controls APP trafficking from endosomes to the Golgi. A reduction in Vps retromer proteins leads to abnormal Vps-SorLA complex that hinders APP trafficking casing APP accumulation in the endosomes where it is subject to beta-secretase. However, mimicking APP phosphorylation at S655, within the APP 653YTSI656 basolateral motif, can enhance APP retrieval in a retromer-mediated process causing decreased APP lysosomal targeting, and decreased Abeta production [43]. An increase in full‐length APP and APP fragment β‐CTF can in turn elevate Rab5 GTPase activity inducing enlargement of early endosome [44]. Interestingly, neuronal atrophy induced by the APP β‐CTFs could be rescued by a dominant‐negative Rab5 mutant both in vitro [39] and in vivo [45].

The net result with aging is depletion of late endosomes, late endocytic dysfunciton, and impaired lysosomal fusion. In AD, endocytosis of Aβ increases into enlarged Rab5 early endosomes. The role of Rab7 in AD is yet to be clearly elucidated as both early upregulation and late downregulation of Rab7 have been observed affecting neuronal health in both directions. As emphasized by Bianchi-Smiraglia et al. [7], total cellular GTP that is measured in homogenates does not allow assessment of free GTP available locally for the Rab GTPases. Free GTP measures have not been reported yet, so their possible role in age and AD-related impairments in endocytosis is unexplored. Therefore, it will be important to measure free GTP in live cells and the effects of variations in free GTP on endocytosis as a function of age and in AD models.

## Common GTPase signaling pathways in autophagy: macroautophagy (mitophagy), microautophagy, and CMA

**Fig. 3:**
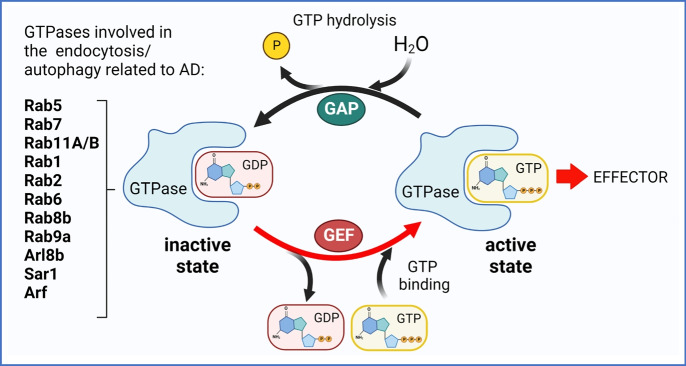
Molecular switch between GTPase states. Small GTPase’s active state (GTP-bound) is achieved by nucleotide exchange factor that catalyzes the exchange of GDP to GTP resulting in GTPase activation. Active GTPase interacts with several kinds of downstream effectors to modulate their activity. GTPase activating protein (GAP) inactivates the GTP-bound proteins by boosting their activity for GTP hydrolysis. The GDP-bound form cannot bind effectors

The GTPase superfamily comprises a wide range of proteins that act as molecular switches by binding and hydrolyzing GTP molecules for tethering, docking, and fusion of vesicles to target membranes [46]. Changes in GTPase function are related to alterations in the trafficking of cargo [47]. The switch between an “active” state (GTP-bound GTPase) and an “inactive” state (GDP-bound GTPase) requires a guanine nucleotide exchange factor (GEF) and GTPase activating protein (GAP) [48] (Fig. 3), termed the Rab GEF/GAP cascade [22]. For many GTPases, the lifetime of the activated, GTP-bound state is believed to serve as a regulator in determining the activation time of a biological event such as membrane fusion and signal transduction. However, proper function of the GTPases could also be limited by a decrease in intracellular GTP levels.

Autophagosome formation involves Rab family members Rab1, Rab5, Rab7, Rab9A, Rab11, Rab23, Rab32, and Rab33B. Rab9 is required in non-canonical autophagy. Rab7, Rab8B, and Rab24 have a key role in autophagosome maturation. Rab8A and Rab25 are involved in unknown aspects of autophagy [22]. Rab11 is required for exocytosis [49]. Failing autophagy is associated with several unhealthy conditions such as metabolic stress and aggregation of proteins associated with neurodegenerative disorders including Alzheimer’s disease. Rab8b has a key role in orchestrating autophagic maturation although its downstream effector Tbk-1, which directly phosphorylates p62 at Ser-403, a crucial residue for autophagic function of p62 [50]. TBK-1 is also required for cytokinesis-induced autophagic elimination of bacteria, whereas Rab8 knockdown after induction of autophagy caused a decrease of phagosomes [50].

Since GDP is tightly bound by Rab GTPases and their intrinsic GTP hydrolysis rates are low despite their high affinity 10^−1^–10^−5^ μM kKm [51], the Rab GEFs catalyze the dissociation of GDP. Rab GAPs facilitate the hydrolysis of GTP. Both regulators are required to coordinate the temporal-spatial activity of Rab GTPases [52]. The activity of Rab GTPases, GEFs, and GAPs are crucial for transport and trafficking of autophagosomes for macroautophagy. Macroautophagy needs stringent control, and some Rab GAPs seem to function in overlapping pathways. For example, TBC domain containing proteins, TBC1D14 and TBC1D15, coordinate endosomal trafficking and autophagosome biogenesis. TBC1D5 is a Rab7 GAP that is recruited to mitochondria by protein FIS1 to handle Rab7 GTP hydrolysis, allowing also mitochondria to regulate the contact untethering with lysosomes [53]. Since mitochondria-lysosome contacts mark the sites of Drp1-positive mitochondrial fission events, alterations in the Rab7 GTP hydrolysis lead to both abnormal lysosomal morphology and markedly reduced the rate of mitochondrial motility [53]. TBC1D2, which affects the Rab7 GTPase and modulates autophagosome-lysosome fusion, was shown to be activated by LRRK1 upon macroautophagy induction [54].

**Fig. 4:**
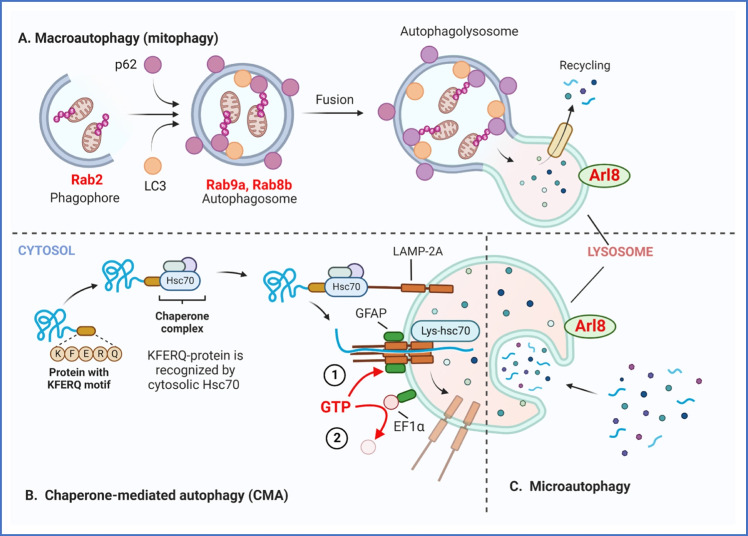
Types of autophagy pathways regulated by GTP **A** In macroautophagy (mitophagy), ubiquitin (Ub)-labeled proteins recruit p62 to interact with LC3 to form autophagosomes. Rab2 participates in phagophore formation, whereas Rab8b and Rab9a participate in autophagosome maturation. Arl8 is located on the lysosome membrane and facilities lysosomal trafficking. **B** In chaperone-mediated autophagy (CMA), substrate proteins bind to the monomeric form of LAMP-2A after recognition by a KFERQ motif by cytosolic Hsc70 chaperon complex. Unfolding of the complete substrate is required for its translocation into a multimeric complex with LAMP-2A. There are two pools of GFAP in the membrane of lysosomes: 1, in conditions with high CMA activity, GFAP interacts with LAMP-2A to stabilize the complex required for the translocation of CMA cargo in a GTP-dependent manner; 2, GFAP interacts with EF1α, while GTP induces the release of EF1α from GFAP inhibiting CMA and promoting the disassembly of multimeric LAMP-2A. **C**. In microautophagy, autophagic cargo are engulfed by invaginations of the lysosomal membrane to capture content. In all of them, recycling occurs after lysosomal degradation

The protein family of small Rab GTPases control vesicle transport routes and ensures trafficking of vesicles to their appropriate target compartments. Rab GTPases interact with effector proteins such as cargo sorting complexes, motor proteins, and tethering factors, which are required for vesicle budding, transport, and fusion of various intracellular organelles. In mammalian cells, there are three primary types of autophagy: macroautophagy, microautophagy, and chaperone-mediated autophagy (CMA) (Fig. 4). Each subtype consists of different mechanisms of substrate delivery to the lysosome; however, the result is the same for all of them culminating in the delivery of cargo to the lysosome for degradation and recycling.

Macroautophagy of mitochondria is the most studied and is commonly referred as mitophagy. Mitophagy signaling is mainly controlled by the Target of Rapamycin protein complex 1 (TORC1). Following induction of autophagy, a sequestering membrane called a phagophore is formed (Fig. 4A). The phagophore encloses misfolded proteins and/or dysfunctional organelles until it is completed into an enveloped autophagosome. The autophagosome then fuses with the lysosome, transferring the cytoplasmic cargo for hydrolysis. The organelles inside the cargo are then degraded into amino acids and simple fatty acids and carbohydrates for release into the cytoplasm by lysosomal/vacuolar membrane permeases and re-use in biosynthesis [55].

CMA (Fig. 4B) does not use membranous structures to sequester cargo, but instead uses chaperones to identify cargo proteins and translocate them to the lysosomal membrane, while microautophagy (Fig. 4C) utilizes invaginations or protrusions of lysosomal membrane to capture and deliver the autophagic cargo to the lysosomal membrane [56, 57].

Reactive oxygen species (ROS) partially controls autophagy. Starvation stimulates ROS (mainly H_2_O_2_) production in mitochondria, which appears to be necessary for autophagosome formation. In yeast, autophagy can be regulated by ROS via Atg4, through oxidation–reduction of a disulfide bond between residues Cys338 and Cys 394, which is required for a proper autophagosome biogenesis [58]. Atg4, a redox protease, acts as a conjugating enzyme cleavage C-terminus in immature Atg8 (mammalian homologue LC3) to expose the conserved glycine residue for its subsequent linkage to phosphatidylethanolamine (PE). Further, Atg4 acts also as a deconjugation enzyme that cleaves the amide bound between Atg8 and PE, which releases it from the membrane for recycling, which is crucial for the conjugation systems essential for autophagy.

## Autophagy: GTP regulation in aging and AD

Autophagic clearance of damaged cellular components or aberrant protein aggregates becomes increasingly important to handle the increased oxidative stress associated with aging and AD [59]. The bidirectional trafficking into and out of cells must be highly coordinated. Autophagy works in association with endocytosis and exocytosis, both of which contribute to turnover of damaged intracellular constituents employing lysosomal fusion and digestion. Autophagy involves tagging defective cellular organelles and protein aggregates for degradation, followed by the assembly of an autophagic structure to transport cargo for fusion with lysosomes to degrade damaged proteins and organelles for recycling amino acids and lipids or disposal. It is a highly dynamic process essential to maintain cellular homeostasis and functions. Dysregulation of autophagy has been linked to aging and pathological neurodegenerative diseases including Alzheimer’s disease. Although it is well known that ATP levels decline with age under pathological conditions [60], age-related changes in GTP levels are poorly elucidated.

Autophagy induction is dependent on ADP/ATP balance. Decreased ATP production stimulates AMP-activated protein kinase (AMPK), and stimulation of AMPK inactivates mTOR. AMPK increases autophagy not only indirectly through inactivation of mTOR but also directly through phosphorylation of Unc-51-like kinase 1(Ulk1) which is the molecular target of mTOR in the autophagic machinery [61].

### GTP depletion may impact autophagy in aging and AD

Post-mortem analysis of AD patients indicates an accumulation of autophagosomes and other prelysosomal autophagic vacuoles in dystrophic neurites and synaptic terminals, which are neuropathological hallmarks of AD [62]. Upregulation of autophagosomes in hippocampal CA1 pyramidal neurons is related to changes in expression of autophagy-related genes (ATG3, ATG5, ATG12, ULK1 and PIK3C3/VPS34) and proteins (LC3B-II and LC3B-I) at early AD stages [63]. These facts suggest that AD is associated with alterations in trafficking of autophagosomes.

Autophagy dysregulation occurs in both AD patients and animal models. Accumulation of large amounts of autophagic vacuoles in neuronal dendrites occurs in PS1/APP double transgenic mice appears even before Aβ plaques [64]. Similarly, immature autophagic vesicles in axons were observed in hippocampal neurons of AD mice, far before synaptic and neuronal loss (Cataldo et al., 2004 [44, 65, 66]. Tau aggregates are also degraded through the autophagy pathway [67, 68]. The wild type presenilin gene 1 (PS1) acts as a ligand of the v-ATPase V0a1 subunit regulating the distribution of v-ATPase subunits into lysosomes for acidification. Intracellular Aβ binds to v-ATPase and inhibits acidification [69]. Mutation in PS1 thus contributes to the dysregulation of the autophagy-lysosome degradation system [70]. The major genetic risk factor for sporadic AD, Apolipoprotein E4 (ApoE4), also contributes to induction of autophagy by lysosomal leakage [71] leading to impaired endolysosomal trafficking, disruption of synaptic homeostasis and reduced amyloid clearance. Altogether this suggests that the defective autophagy-lysosome proteolysis pathway might be responsible for the accumulation of pathogenic proteins such as Aβ and tau in AD. A conditional knock out of a gene needed for autophagosome formation, Atg7^*flox/flox*^, crossed with APP23 transgenic mice, indicated that impaired autophagy deficiency promoted Aβ accumulation in CA1 and cortical pyramidal neurons, as well as a drastic reduction in the extracellular Aβ plaque burden and inhibition of Aβ secretion [72]. This observation suggests that alterations in autophagosome formation avoids proper Aβ processing that could lead an aberrant accumulation in the soma [27]. Aβ accumulation has been also observed in the cis- and trans-face of the Golgi vesicles in the late Golgi apparatus, indicating this organelle could also produce functional alterations that impair the proper formation of the phagophore [73]. Supporting this supposition, the small GTPase Rab2 connects the Golgi network to the autophagy pathway machinery [74]. Rab2 participates in the formation of phagophores by further recruiting and activating Ulk1. Rab2 interacts with Rubcnl and Stx17 (an autophagosomal SNARE protein) to further specify the recruitment of HOPS complex to facilitate autophagosome maturation and fusion with lysosomes [74].

**Fig. 5:**
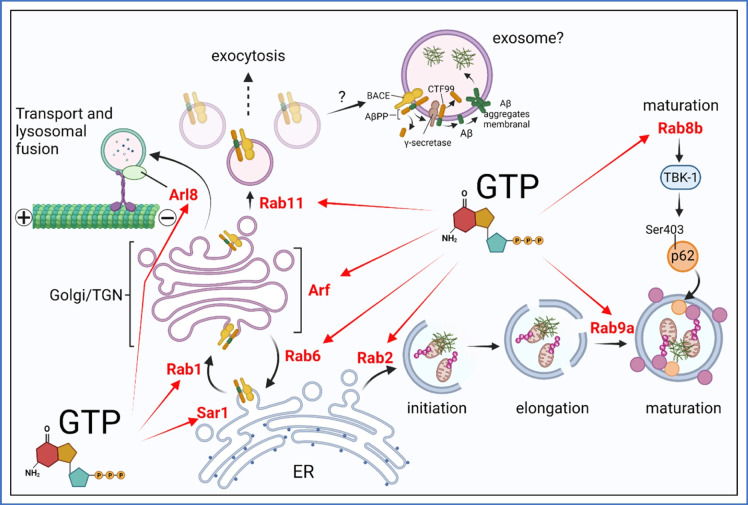
Autophagy requires the participation of several small GTPases. In the initiation phase, Rab2 recruits and activates ULK1 in the phagophore formation, while Rab9a and Rab8b participate in autophagosome maturation. Arf takes part in budding in the Golgi complex, and recruitment of coat proteins during vesicle formation. Rab1 participates in the bidirectional vesicular transport route between the endoplasmic reticulum (ER) and Golgi apparatus. Sar1 GTPase is involved in the COPII-mediated transport as the exit route of the APP once its final conformation has been reached. Rab6 resides in the TGN and participates in retrograde transport from Golgi to ER, and has been associated with the regulation of the vesicular transport and processing of APP. Rab11 controls the endosome recycling to the plasma membrane. Disruptions in vesicular export that contains APP-BACE could cause an accumulation of exosomes. Arl8 regulates the transport and lysosomal fusion via microtubules along the neuron

Another member of Ras super family of small GTPases involved in vesicle formation is the ADP-ribosylation factor (Arf). Arf GTPase mainly participates in the budding process in the Golgi complex, recruitment of coat proteins during vesicle formation for membrane trafficking. Arf GTPase is involved in control of APP trafficking through MINTs proteins, crucial components for the fusion of synaptic vesicles. MINT proteins bind directly to Arf GTPases and co-localize with APP-containing vesicles to regions of the Golgi/trans-Golgi network (TGN), with intracellular APP levels proportional to MINT levels [75]. The knockdown of Arf1 decreased secretion of amyloid peptides [76], suggesting the impact of failures in Arf1 function on trafficking of APP, which could converge in intracellular accumulation (Fig. 5).

Dysregulation of various Rab proteins also contributes to AD pathology. Rab1 dysregulation induces fragmentation of the Golgi apparatus, which triggers hyperphosphorylation of tau by activation of cdk5 and ERK1/2 [77, 78]. A defect in Rab6 can influence the secretion of APP into the medium, leading to alterations in the secretion rate, which could promote changes in the anterograde trafficking of APP leading to intracellular accumulation. Supporting intracellular accumulation, an exome sequencing analysis identified Rab11A/B as a component of late-onset AD risk. Rab11 controls the endosome recycling to the plasma membrane. Silencing Rab11A/B in primary neurons isolated from APP transgenic mice reduced Aβ levels in the supernatants collected and analyzed by electrochemiluminescence (ECL) [79]. These data suggest that the accumulation of Aβ could also be generated by failures in the export of APP-containing vesicles, which could give way to a cleavage by the secretase in the vesicular membrane [27] (Fig. 1B). Since formation of the APP-containing vesicles and the formation of the phagophore involve a correct functioning of the ER-Golgi driven by small GTPases, deficits in GTP levels would cause disturbances in the packaging of APP that could compromise its secretion and promote intracellular accumulation.

**Fig. 6:**
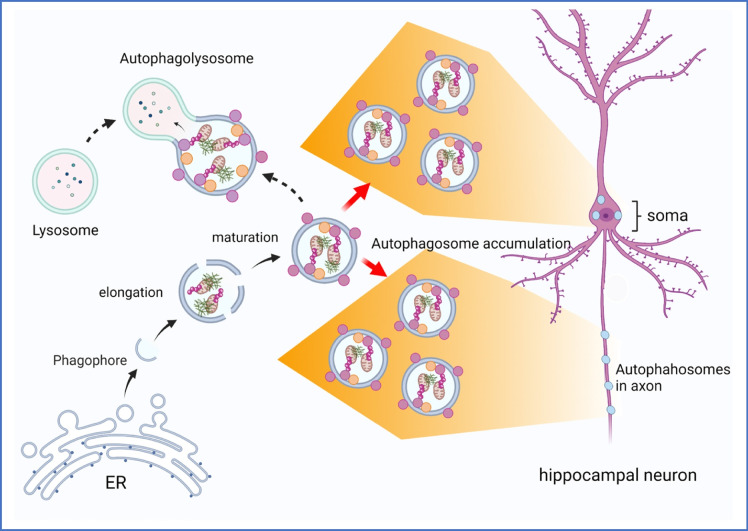
Deficiency in autophagolysosome formation in AD. Mature autophagosomes (p62 and LC3 positive cells) containing Aβ aggregates are accumulated in the soma and axons leading to vesicular imbalance further associated with energy failure

These relationships of autophagy to accumulation of intracellular Aβ are supported by an age-related colocalization signal between p62-directed formation of autophagosome and Aβ forms observed in primary cultured hippocampal neurons from adult 3xTg-AD mice [27] and in APP23-transgenic mice with Atg7 floxed mice [73]. Another interesting fact is that no signal of aggregates was observed in autophagolysosomes positive for cathepsin D [27]. These data may indicate dysfunction in earlier steps in autophagy or in lysosomal function in AD model neurons (Fig. 6) [26].

### GTP regulation in CMA in AD

Instead of organelles, CMA degrades small molecules in eukaryotic cells induced by prolonged starvation or mild oxidative stress. CMA has been proposed to be regulated by GTP levels [80]. This regulation involves the participation of the intermediate filament glial fibrillary acidic protein (GFAP) and the elongation factor-1 alpha (EF1α) [46, 81]. GFAP is present in two different pools at the lysosomal membrane, a portion bound to LAMP-2A and another unbound form that interacts with EF1α. The three KFERQ motifs in the Hsc70 chaperon direct it to lysosomal membranes where it interacts with protein complex LAMP-2A and stabilizes the translocation of CMA-cargo into the lysosomal lumen. The portion of GFAP not bound to LAMP-2A contributes to GTP regulation. In the presence of GTP, EF1α is released from GFAP at the lysosomal membrane which promotes the dissociation between GFAP and LAMP-2A, mobilizing LAMP-2A to the lipid microdomains for its degradation and subsequent CMA inhibition [81]. Failures in LAMP2A complex density on lysosomal membrane are also associated with aging, which also leads to a decrease in CMA function [82] (Fig. 4B).

Yet another form of mammalian autophagosome biogenesis operates through an enigmatic non-canonical VPS34-independet pathway. Phosphoinositides (PIs) define the membrane identity and control several membrane trafficking events. Phosphatidylinositol 5-kinase (PIKfyve) converts endosome-localized phosphatidylinositol-3-phosphate (PI(3)P) to PI(3,5)P2, a key regulator of early to late endosome membrane trafficking [83]. Further, PIKfyve complex is also responsible for production of PI(5)P from PIs and regulates autophagosome formation. PI(5)P regulates autophagy via PI(3)P effectors (recruiting WIPI2 and DFCP1 proteins), which provide a mechanistic framework for this alternative autophagy pathway. PI(5)P is used by phosphatidylinositol 5-phosphate 4-kinase β (PI5P4Kβ) that regulates PI(5)P levels using GTP rather than ATP for PI(5)P phosphorylation to obtain PI(4,5)P2 as a final product that regulates actin cytoskeleton remodeling [84]. They found PI(3,5)P2 has low affinity for cofilin, which disassembles actin filaments and high affinity for N-WASP, which activates Arp2/3 complex to initiate actin nucleation to transport endocytic vesicles from the plasma membrane. PI5P4Kβ activity is proposed to reflect changes in direct proportion to physiological GTP concentration, acting as an intracellular GTP-sensor [85]. In cells lacking PI3P (low PI(3,5)P2) with locked VPS34, the PIKfyve complex sustains autophagy through the use of PI5P [85]. These data indicate PIKfyve has a pivotal role in the modulation of autophagy. Impaired PIKfyve function drives formation of swollen vacuoles, easily visible at low magnification in living cells [86]. The intracellular domain of APP binds the Vac14 subunit of PIKfyve complex, affecting PI(3,5)P2 production [87]. PI(3,5)P2 binds and activates the endolysosomal TRPML channel [88]. They found enlarged vacuoles in PI(3,5)P2-deficient mouse fibroblasts that were suppressed by overexpression of healthy TRPML1 channel. TRPML conductivity and lysosomal acidification were impaired [89].

## Dependence of mitochondrial fission and fusion on GTP

**Fig. 7:**
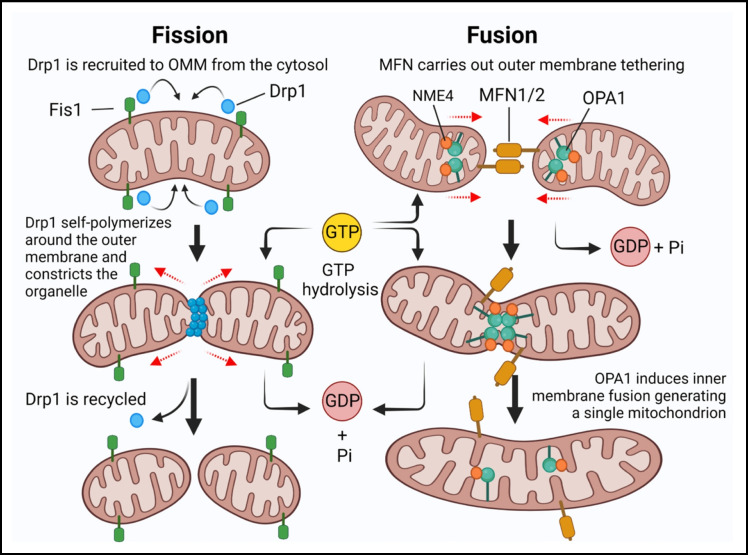
GTP-dependent mitochondrial dynamic morphology of fission and fusion. Left fission panel: Dynamin-related protein-1 (Drp1) executes the mitochondrial fission by self-polymerizing around the outer mitochondrial membrane constricting and severing both membranes in a process dependent on GTP hydrolysis. Right fusion panel: Mitofusin-1 and -2 (MTF1/2) tethers the adjacent outer mitochondrial membranes in a process dependent on GTP hydrolysis. OPA1 enables inner mitochondria membrane fusion using local GTP provided by NME4. Red dashed arrows indicate the displacement of the organelle

In addition to providing ATP through oxidative phosphorylation, mitochondria also provide GTP from NME4 and its nucleoside diphosphate kinase activity [8]. This mitochondrial supply of energy is essential for generation of synaptic vesicles for release at axon terminals as well as for vesicular recycling at synapses. Synaptic loss in AD could be caused by loss of bioenergetic capacity to maintain these essential processes. Therefore, the number and localization of mitochondria to synapses likely determines the energetic capacity for endocytosis and exocytosis. Mitochondrial dynamics delicately balance fission and fusion controlled by Drp1 and Fis1, Mf1, Mfn2 and Opa1 [90, 91] (Fig. 7). ATP conversion to GTP is locally controlled by localized nucleoside diphosphate kinases (NDPKs) from the NME genes 1–4 [51]. NME1 and 2 (sometimes called NM23 H1 and H2) are predominantly cytosolic, while NME3 and 4 (NM23 H3, H4) are mitochondrial. The mitochondrial NDPKs complex with specific dynamin GTPases to channel GTP directly from ATP hydrolysis [16]. The balance of mitochondria fission and fusion is sensitive to redox imbalance. Either endogenous or exogenous application of ROS activates mitochondrial fission, inducing mitochondrial fragmentation and subsequent mitochondrial dysfunction [90]. This leads to further ROS overproduction and a vicious cycle that amplifies oxidative stress and ultimately causes oxidative imbalance in AD [92]. Fission is regulated by the dynamin GTPases Drp1 and Fis1 with Km’s around 100 μM [51]. They polymerize and constrict tubular membranes much like endocytosis. Fis1 is localized in the outer mitochondrial membrane [90, 93, 94].

Fusion is controlled by three GTPase proteins: Opa1, located in the inner mitochondria membrane and Mfn1 and Mfn2, located in the outer mitochondrial membrane. The Opa1 GTPase with a Km around 500 μM would require abundant levels of GTP to polymerize and mechanize mitochondrial membrane fusion into tubes [51].

Mitophagy can be activated by several stimuli such as hypoxia, energetic stress, and increased oxidized redox state. Increased oxidative stress and elevated ROS levels caused fragmentation of mitochondria and induction of DRP1 fission-dependent mitophagy in mouse and HeLa cells. This did not result in cell death and autophagy because moderate levels of ROS were not sufficient to trigger non-selective autophagy [95]. Indeed, this mitophagy can be inhibited by N-acetyl-l-cysteine through fueling of the glutathione pool and possible action on Atg4. A decrease of the glutathione pool also induced mitophagy but not general autophagy. Conversely, the addition of a cell-permeable form of glutathione inhibited mitophagy [96]. Thus, an oxidative redox state promotes target-selective removal of dysfunctional mitochondria. This also suggests integration of redox balance and energy levels to maximize healthy mitochondrial function and control turnover of damaged mitochondria.

Impairment in fusion and fission has been implicated in AD. In a mouse model, Aβ interacts with fission protein Drp1, with a subsequent increase in free radical production, which further activates Drp1 and Fis1, causing excessive mitochondria fragmentation, defective transport of mitochondria to synapses, lowers synaptic ATP, and ultimately leads to synaptic dysfunction [97]. p-tau also interacts with Drp1 and enhances GTPase Drp1 enzymatic activity, leading to excessive fragmentation of mitochondria and mitochondrial dysfunction in AD [98]. A Drp1 S-nitrosylation adduct (SNO-Drp1), further stimulated its activity, and led to excessive mitochondrial fragmentation, and synapse loss [99].

## Impaired lysosomal function in AD caused by age-related energy depletion

Lysosomal digestion of autophagic cargo is the last step for the completion of autophagy. Therefore, lysosomes must maintain its acidic milieu for pH-based degradation of cargo by acid-activated peptidases, lipases, nucleases, and glycosidases. For lysosomal acidification, the influx of protons is carried out by both the v-ATPase, an ATP-dependent proton pump and chloride proton antiporters, while cation efflux is mediated by transporters TPC and TRPML, which are also involved in the pH balance [100]. Presenilin-1 (PS1) regulates the distribution of v-ATPase subunits into lysosomes acting as a ligand of the v-ATPase V0a1 subunit and maintains lysosomal homeostasis via TRPML1 [69]. PS1 mutations have been linked to low lysosomal acidification, dysregulation of the autophagy-lysosome degradation system and the pathogenesis of early onset AD [26].

**Fig. 8:**
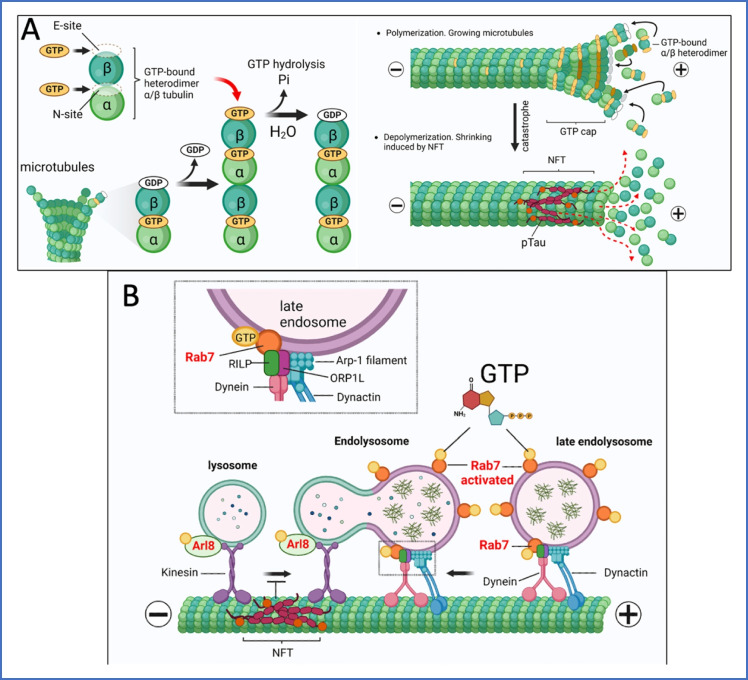
Dynamic instability of microtubules associated with GTP. **A** GTP molecules bind to E-site and N-site on α/β heterodimer tubulins. GTP at the E-site is hydrolyzed to become GDP-β-tubulin and exchanged for a new GTP-bound heterodimer α/β to assemble subunits. Heterodimers are added to the growing microtubule lattice for polymerization forming a new layer of GTP-heterodimers known as a GTP-cap. Depolymerization occurs when heterodimers leave the shrinking microtubules lattice. Hyperphosphorylation of microtubule associated protein, tau promotes neurofibrillary tangle (NFT) formation and microtubule destabilization. The transition from a growing state to a catastrophic shrinking state. **B** Endolysosome formation requires lysosomes move along microtubule tracks in the positive direction, while late endosomes move in the negative direction. The lysosomal multiprotein complex BORC (not shown) activates the small GTPase Arl8 to engage kinesin-driven plus-end transport. For minus-end transport, the Rab7-GTP-bound state recruits the Rab-interacting lysosomal protein (RILP) and the cytosolic oxysterol-binding protein-related protein 1 (ORP1L) forming the dynein-dynactin complex. NFT formation disrupts vesicular traffic along microtubules

Arl8 (an Arf-like G protein) is a small GTPase located on lysosomes that acts as a linker between lysosomes and kinesin-1 to facilitate lysosomal trafficking along axons (Fig. 8B) [101]. Arl8b also acts as a switch to regulate the association of HOPS complex with the lysosomal membrane [102]. Disruption of Arl8b function causes abnormal accumulation of cholesterol in the membranes of lysosomes driving impaired axonal lysosome trafficking and leading to autophagic stress and axonal autophagosome accumulation [103]. Additionally, elevated Arl8b expression rescued lysosome transport into axons and autophagic stress. Overexpression of Arl8 also stimulated the bidirectional motility of lysosomes on microtubules by binding to the kinesin-1 linker SKIP to link kinesin and power motility [104]. SKIP required Arl8 in its active GTP-bound state for binding. However, overexpression of Arl8b also caused a striking alkalinization of lysosomes and movement toward the cell periphery versus a more uniform distribution of lysosomes throughout the cytoplasm [105]. A proteomic study in human tissue reported enrichment of Arl8b in amyloid plaques [106]. These data suggest that changes in the expression or functioning of Arl8 affects the fusion of the lysosome with the autophagosome or late endosome, which could have implications in autophagosome accumulation observed in AD. Since formation of secretory vesicles delivered to the plasma membrane is a GTP dependent process, requiring Arf and Rab GTPases, deficits in GTP levels may compromise proper vesicular trafficking.

Lysosome biogenesis is regulated by mTORC1 and the transcription factor EB (TFEB) creating a reversible signaling complex on the lysosome surface. mTORC1 phosphorylates TFEB on Ser211, driving thereby enabling TFEB transport to the nucleus to upregulate v-ATPase expression and other genes involved in lysosome biogenesis and autophagosome formation [107, 108]. Addition of Aβ to microglial cells lowers TFEB in the nucleus and impairs processing of Aβ [109]. This regulation by mTORC1 is in turn supported by small GTPases including Rheb and Rag, amino acid-sensing components included in the multiprotein signaling complex regulator which acts as an activator of mTORC1 [110]. Since lysosomal acidification requires ATP for vATPase function and GTP for GTPase-mediated regulation of TORC1, we hypothesize that energy depletion due to aging or AD-like pathological conditions directly impairs lysosomal function. Additionally, considering that lysosomal acidification involves a nutrient intake-mediated regulatory interaction between v-ATPase and TORC1 via TFEB, cycles of fasting and nutrient consumption could benefit lysosomal function in AD [111], while frequent sugar consumption may impair function. Impaired lysosomes could lead to accumulation of autophagosomes full of damaged mitochondria and enriched with Aβ-aggregates incapable of being degraded. Since autophagy carries out the replacement of damaged or aged organelles, its maintenance is affected by metabolic changes due to age. Age-related metabolic shifts and impaired lysosomes could lead to accumulation of metabolic shifts such as energy depletion and/or oxidative redox shifts Thus, GTP levels could be lower in low-activity sedentary lifestyles, which would lead to inefficient pathways of GTP-dependent protein degradation with advancing age. These observations strongly suggest a relationship between alterations in the maturation process of the autophagosome and the metabolic deficiencies that occur with the progression of the disease and ageing. Further, aberrant Aβ accumulation could be the consequence of upstream deficiencies in GTP that impair autophagic processing of Aβ and tau, possibly earlier than aggregate amyloid secretion, inflammation and plaque buildup.

## GTP-dependence of tubulin assembly, vesicle transport and protein synthesis

Microtubules are key players in axonal growth and provide structural support to axo-dendritic vesicular trafficking. Microtubule ends undergo a repeated processes of polymerization/depolymerization, called dynamic instability. Microtubule polymerization requires the addition of GTP-bound α/β heterodimers to their ends (Fig. 8A). Tubulin heterodimers bind to two molecules of GTP at two separate sites. The N-site is located at the intradimer interface, between α- and β-tubulin at which GTP is not hydrolyzed and exchanges at a slow rate. The E-site is at the intradimer interface formed by the β-subunit of one heterodimer and the α-subunit of a neighboring heterodimer. GTP at the E-site is hydrolyzed to GDP and exchanged for a new GTP nucleotide. Since microtubules exhibit a high rate of dynamic equilibrium between polymerization states, cytoplasmic levels of GTP must be high enough to power the demand of the dynamic instability in the microtubes (Fig. 8A).

Microtubules form the cytoskeletal tracks on which lysosomes travel to their target endosomes and phagosomes (Fig. 8B). The kinesin motor powers transport of the lysosomes toward the positive-end of axonal microtubules with ATP, but the connection of the kinesin motor to the lysosome requires Arl8 GTPase with bound GTP [101]. Conversely, transport of late endosomes on microtubules toward lysosomes requires Rab7 GTPase and bound GTP to attach the dynein motor.

In AD, the microtubule-associated protein tau polymerizes with hyperphosphorylation into insoluble filaments of axo-dendritic neurofibrillary tangles (NFT) [112, 113]. Once hyperphosphorylated, tau loses it affinity for the microtubules. Wide-ranging studies have approached the alterations in microtubule dynamics, but have failed to identify a clear cause of failure in tau function. Regulation of tau involves GTPases. In AD brains, Rac1 GTPase protein levels decline 50% [114]. In vitro, Rac1 GTPase activation caused hyperphosphorylation of tau_181_, increased Abeta42 production and decreased actin stability in spines [114]. This group also saw a biphasic rise in Rac1 GTPase in young 3xTg-AD hippocampus, followed by a later decline, as in late-stage human AD brains. In another study of tauopathy mice, increased farnesyl transferase activity to translocate the Rhes GTPase to autophagic vesicles was associated with low tau hyperphosphorylation, while inhibition of Rhes promoted tau hyperphosphorylation [115]. Thus, GTPase-dependent autophagy effectively clears pTau [116] and impaired autophagy from low GTP levels or oxidative redox state would impair clearance of pTau.

GTP is also essential for protein synthesis, the synthesis side of proteostasis, that requires the hydrolysis of two GTP molecules for each amino acid incorporated into a polypeptide. While ATP is used for charging aminoacyl-tRNA, for RNA helicase and recycling GDP to GTP, GTP itself is essential for the activity of elongation factors at intitiation (IF-2), elongation (EF-Tu, EF-G) and ribosome release factor for termination (RF1) [117]. This group used a cell-free reconstituted system to measure an overall Km for ATP of 27 µM and 14 µM for GTP. These high affinities suggest prioritization of protein synthesis during energy limitation to maintain high capacity, perhaps at the expense of endocytosis and autophagy which likely have higher Km’s. Consequently, a large amount of GTP is required at synaptic plasticity where metabolic demands are the highest to maintain ionic homeostasis for synaptic function and protein turnover. Protein synthesis is crucial for presynaptic neurotransmitter release as well as consolidation of post synaptic plasticity [118]. Nevertheless, protein synthesis was decreased along with ribosomal RNA and tRNA levels, while RNA oxidation increased in the early phase of AD [119]. Altered protein synthesis leads to a constant accumulation of oxidized proteins leading to misfolding and aggregation. Protein aggregation in turn impairs the activity of cellular proteolytic systems resulting in further accumulation of oxidized proteins [120]. Thus, a vicious cycle results in excess protein ubiquitination and dysproteostasis. Several other genes that encode ribosomal proteins are abnormally regulated leading to altered protein levels of elongation factors eIF2α, eIF3η and eIF5 in AD [121]. Increased eIF2α and decreased eIF3η and eIF5 levels were observed in the hippocampal CA1 region of AD brain. Persistent eIF2α phosphorylation at Ser51 through prolonged overactivation of regulatory kinases inhibits the delivery of initiator methionyl‐tRNA preventing the general translation initiation of a subset of mRNAs. Thus, proteostasis is could be impaired at severely limiting GTP concentrations.

## Impaired GSH-Trx system as a trigger of autophagy failure in aging and AD

Age is the major risk factor in AD and is associated with an imbalance between a decrease in redox buffer protection and an oxidative shift that increases ROS production. However, the proposal that ROS damage is a causal factor in the pathogenesis of AD has not been validated by antioxidant therapy and an oxidative redox shift appears to be upstream of ROS damage [122]. An epigenetic oxidized redox shift (EORS) has been proposed to precede ROS-mediated oxidative damage to account for lasting metabolic alterations in aging and AD [123]. This manifests as a decrease in reductive intracellular redox ratios from the critical systems that maintain redox homeostasis: cysteine/cystine, GSH/GSSH and NAD(P)H/NAD(P) [3]. Across the age-span of non-transgenic mouse brains, an oxidative glutathione redox state precedes an Akt metabolic shift and old-age buildup of aggregated proteins which are greatly accelerated in the 3xTg-AD mouse model before intracellular hippocampal accumulation of Aβ or extracellular plaques [124]. Mutations in enzymes related to GSH metabolism have also been associated with the regulation of autophagy such as glutathione reductase gene (grs-1) in a C. elegans model that abolishes the nuclear translocation of HLH-30 transcription factor (orthologue of mammalian TFEB) and triggering reductions in the transcription of genes related to clearance of protein aggregates by autophagy [125]. Subcellular free NADH concentration decreases with age in mitochondria, nuclei and cytoplasm in live wild-type and AD-like neurons [2]. Could this redox shift stimulate the logarithmic increase in intracellular Aβ-aggregates with age [27]?

Although it seems evident that some combination of an increase in the rate of formation of intracellular Aβ or their rate of autophagic clearance decreases with the age (the main risk factor), the mechanisms that trigger these alterations remain unclear. We propose that the redox state of cells and possibly a systemic redox shift are an essential driver of aging [123]. GSH and thioredoxin (Trx) are the most important thiol redox system in the cells against oxidative stress. However, their distribution in the cell is very different. The physiological GSH concentration is in the range of mM, ~ 1000-fold higher than Trx (in the μM range). Despite this, the Trx system regulates a broader range of proteins than GSH system [126]. Proteomic analysis identified involvement of Trx in the regulation of pathways mainly linked to glycolysis/gluconeogenesis and cytoskeletal remodeling, while pathways affected by both Trx and GSH are related to insulin regulation of translation, lipid metabolism and cell adhesion [126]. In the same study, Trx was also associated with regulation of other proteins involved in handling GTP and autophagy, such as (i) the IQ motif contained in GTPase activating protein 1 (GAP), a scaffolding protein that integrates Rho GTPase and Ca2 + /calmodulin signals in the maintenance of cytoskeletal integrity; (ii) GDP dissociation inhibitor-1 (GDI-1), a protein that maintains Rab proteins in the GDP-bound conformation; (iii) ubiquitin-activating enzyme E1(UBA1), a protein required for Atg7- and Atg3-independent autophagy [127]; and (iv) cofilin-1, an actin-depolymerizing factor whose function is crucial for maintaining synaptic spines and instigating mitochondrial fission and mitophagy [128]. Interestingly, cofilin-1 levels are elevated in AD model mice and human AD, indicating its unbalanced regulation [129]. These data suggest that Trx could have a possible role in maintaining and handling of GTP and autophagy.

The N-terminus of the Rab5 and Rab7 GTPases contain single or double cysteines that should be subject to oxidation–reduction to cystine or farnesylation to anchor in membranes or nitrosylation, perhaps as these Rabs aggregate. Other redox-sensitive proteins in endocytosis such as TXNL1 [130] could accelerate or impede transitions between steps in the pathway.

The low redox potential in the disulfide bond in Atg4 is efficiently reduced and activated by Trx, suggesting a role as a redox regulator of autophagy mediated by Atg4 [131]. Other components in the thioredoxin system are involved in autophagy regulation. Deletion of thioredoxin reductase-2 (TrxR2, mitochondrial isoform) caused mitochondrial degeneration accompanied by overexpression of LC3, p62, LAMP1, and accumulation of autophagic bodies in cardiomyocytes [132]. But a TrxR1 deficiency enhanced oxidative stress, interrupted early autophagy and decreased protein-degradation in lysosomes [133]. These data suggest that several key proteins of the autophagy machinery, such as Atg in yeast, may be regulated in response to changes of intracellular redox conditions, in addition to potential regulation by GTP levels.

## Boosting energy to rescue AD pathology

While stimulation of the overall autophagic flux is beneficial for cell and tissue fitness, without sufficient energy to execute autophagy, the system will be inhibited. Increasing evidence shows that a decrease in NAD^+^/NADH availability with age [2, 134] plays a critical role in age-related neurovascular and cerebromicrovascular dysfunction [135, 136]. Thus, rejuvenation of cellular oxidative and reductive energy may benefit the age-related increased demands of autophagy. Restoring cellular NAD^+^ and NADH levels by nicotinamide mononucleotide (NMN) supplement in aged mice rescued neurovascular function, increased cerebral blood flow, and improved performance on cognitive tasks [135, 136]. Nicotinamide riboside (NR), a precursor to NMN, improves mitochondrial and adult stem cell function in aged mice as well as extend overall lifespan [137]. These NAD precursors can also alter autophagy. NR can prevent the blockage of autophagic flux and reduce oxidative stress in doxorubicin-treated cardiomyocytes, leading to enhanced autolysosome clearance via the NAD^+^/SIRT1 signaling pathway [138]. NR treatment also increased autophagic function and restored mitochondrial health in animals models of Parkinson’s disease, suggesting possible treatments in other neurodegenerative diseases [139]. Since the product of Sirtuins and PARP is nicotinamide, cells need the NAD salvage pathway to recycle nicotinamide back into NMN and ultimately NAD^+^. Two labs have treated 3xTg-AD mice with nicotinamide and observed beneficial improvement in memory, DNA repair, autophagy, accumulation of Aβ, and phosphorylated tau and improved bioenergetics [140, 141]. Similarly, an improvement in cognitive functionality on multiple behavioral tests in 3xTgAD mice treated with nicotinamide riboside suggests a pivotal role for cellular NAD + depletion upstream of neuronal degeneration in AD [142]. Therefore, we treated adult 3xTg-AD and non-transgenic neurons with nicotinamide to see that the bioenergetic improvement extended to improvement in GTP levels, both bound and free (Fig. 1). Overall, increased NAD^+^ levels may benefit numerous downstream functions such as NAD-dependent SIRT1 activated genes which rejuvenate mitochondria and inhibit inflammation and apoptosis.

Another class of molecules benefitting old neurons are Nrf2 activators [143, 144]. One example is the ester of epigallocatechin and gallic acid, ( −)-Epigallocatechin-3-Gallate (EGCG), the most bioactive polyphenol found in green tea extract. EGCG significantly increased mRNA expression of the key autophagy adaptor proteins NDP52 and p62 and enhanced the clearance of AD-relevant phosphorylated tau species in primary neurons [145], as well as improving cerebrovascular tone [146]. EGCG also activated autophagic pathways by inducing Sirt1, exerting protective effects against human prion protein-induced neurotoxicity [69]. Similar effects were observed using other Nrf2 electrophiles sulforaphane [147], fisetin and urolithin A from pomegranate [148].

## Conclusion and future studies

A surprising array of specific GTPase control macroautophagy (mitophagy), microautophagy, chaperone-mediated autophagy (CMA), and endocytosis and vesicle and mitochondrial trafficking. Since they all depend on local GTP levels, it will be important to determine their binding constants for GTP. Zala et al. [51] demonstrated functional significance for mitochondrial fission GTPases Drp1 and Fis1 with Km around 100 μM, while the Opa1 GTPase with a Km around 500 μM, would require higher GTP levels to promote mitochondrial fusion. In AD, the endocytic and exocytic processing of the synaptic adhesion protein beta-APP may be impaired by lower local GTP levels and GTPases as well as local oxidative redox states. More clear in AD is the accumulation of autophagic vesicles from either blockages in the long pathway or failure to upregulate a sufficiently robust response to internal cellular damage coupled with failure to sufficiently acidify lysosomes for activation of cargo degradation. Less clear is how much of these deficits are caused by age-related degeneration, known to be associated with age-related oxidative redox shifts. Age-related changes in GTP levels or capacity to upregulate bioenergetic fluxes will be an important aspect of future investigation, especially if NAD^+^ precursors are able to boost capacity and promote more autophagic clearance. The metabolic adaptation to a sedentary lifestyle could downregulate mitochondrial function with less energy for healthy management of amyloid and tau proteostasis, synaptic function, and inflammation. In subjects with deficits, certain energy precursors and redox modulators may add to the benefits of exercise and a healthy diet to extend and promote maximum healthspan.

## Data Availability

Raw image data for Figure 1 is available on reasonable request to the corresponding author.
